# Evaluating the impact of China’s MSW sorting pilot policy on urban sustainable development: Empirical evidence from 95 cities

**DOI:** 10.1371/journal.pone.0296819

**Published:** 2024-02-20

**Authors:** Qi Mao, Xiaojun Jia, Jingcheng Li, Tianyang Wang

**Affiliations:** 1 School of Public Finance & Economics, Shanxi University of Finance and Economics, Taiyuan, China; 2 Beijing Laboratory of National Economic Security Early-warning Engineering, Beijing Jiaotong University, Beijing, China; 3 School of Economics and Management, Beijing Jiaotong University, Beijing, China; National Technical University of Athens: Ethniko Metsobio Polytechneio, GREECE

## Abstract

The escalating challenge of municipal solid waste (MSW) critically tests the sustainable development capacities of urban centers. In response, China initiated pilot policies in 2017 aimed at bolstering MSW management. The effectiveness of these initiatives, however, necessitates empirical scrutiny. This study leverages panel data spanning 95 cities at the prefectural level or higher, covering the period from 2006 to 2020, to assess the impact of the MSW sorting pilot policy on urban sustainable development using a difference-in-differences approach. The research found that the MSW sorting pilot policy has significantly increased the processing volume of MSW, thereby enhancing the sustainable development capabilities of cities. Further, the study identifies augmented fixed asset investments as a key mechanism through which pilot cities have enhanced their MSW management capabilities. Notably, the policy’s stimulative effects are more pronounced in less densely populated and economically lagging regions. These findings provide critical insights for developing nations in shaping MSW sorting strategies and advancing urban sustainability.

## 1. Introduction

Proper municipal solid waste disposal is fundamental for the long-term health, prosperity, and sustainability of cities and their residents [1]. High-income countries have implemented comprehensive urban waste management systems. In contrast, low- and middle-income countries often grapple with challenges such as limited funding and lower environmental awareness, posing barriers to establishing effective and comprehensive mechanisms for urban household waste management [2].

In 2004, China ascended as the global epicenter of Municipal Solid Waste (MSW) production, culminating in marked environmental challenges, notably affecting water, air, and soil quality [3]. At present, 220 urban centers grapple with the predicament of being encircled by waste [4]. While MSW indisputably impacts the environment detrimentally, its judicious recycling and reuse can channel renewable resources. Furthermore, adept management of MSW holds the potential to bolster the quality of life for urban dwellers [5].

Waste generation in developing countries is increasing rapidly [6], and the effectiveness of their waste management policies still needs improvement. The need for more MSW processing capacity means that developing countries commonly face challenges related to inadequate waste management capabilities [7]. If appropriate guiding policies are introduced, their waste processing abilities can be improved in the short term; for example, proper waste sorting is a crucial means to enhance MSW processing capacity.

In 2017, China launched an initiative for MSW sorting, led by the State Council’s directive. This program, focusing on 46 key cities including Beijing, Tianjin, and Shanghai, aimed to develop a model for waste segregation. The approach involved increasing public awareness of waste classification, boosting investment in MSW treatment facilities, and refining the framework for waste collection, transportation, and processing. The MSW classification pilot policy in China, exemplified by Shanghai’s approach, encompasses four main aspects. Firstly, it mandates that public buildings and facilities be equipped with garbage collection systems, with new constructions required to integrate these systems into their primary design. Secondly, the policy emphasizes strengthening the recycling system infrastructure, including transportation and processing equipment, under governmental oversight. Thirdly, it encourages collaborative participation in waste sorting among residents, property service enterprises, and homeowners. Lastly, the policy advocates for widespread societal engagement, incorporating waste classification into school curricula and establishing educational bases for public awareness on waste management.

In recent years, as environmental awareness has grown in developing countries, scholars have been conducting more research on the role of waste management policies. Li et al. believe that waste sorting policies can reduce MSW on-site and effectively enhance waste disposal efficiency [8]. Azevedo et al. (2021) conducted a comparative analysis of waste management cases in Germany and Brazil, concluding that clear legal rules, regular publicity, and charging fees benefit waste management. Andersson and Stage (2018) analyzed Sweden’s waste policy’s direct and indirect impacts on waste sorting behaviors [9]. Adriyanti et al. (2018) take Indonesia as an example; they believe that improving the waste management system mainly relies on community participation instead of increasing fixed asset investment [10]. However, Ddiba et al. (2020) argue that increasing investment in urban infrastructure construction can significantly improve the level of MSW management and enhance the capacity for sustainable development [11]. Some scholars also believe that to establish an efficient MSW collection system in developing countries, emphasis should be placed on environmental education activities [12]. Increasing evidence indicates that enhancing the sustainable development capacity of cities requires the concerted efforts of governments, communities, stakeholders, and citizens [13,14].

Existing literature extensively discusses various factors that impact MSW processing capacity, and most scholars are inclined to formulate specific policies to enhance MSW processing capabilities. However, there still needs to be more discussion regarding the effectiveness of specialized MSW (Municipal Solid Waste) sorting policies in developing countries. This lack of focused discourse leads some nations to formulate MSW-related policies without a clear policy focus, preventing them from maximizing the use of limited administrative resources to enhance the sustainability of urban development.

The main innovative points of this article are as follows: First, the quasi-natural experimental method is used to validate the role of MSW sorting pilot policies in enhancing urban sustainability. Second, mechanism analysis clarifies the mediating factors by which the pilot policy enhances local MSW processing capabilities. The findings above can provide a more precise direction for MSW policy formulation in other countries.

## 2. Materials and methods

### 2.1 Study area and data source

This study’s sample comprises panel data from 95 cities spanning from 2006 to 2020. Among these, 46 cities implementing MSW sorting pilot policies were designated as the experimental group. Due to constraints in data availability, 49 other cities that provided relevant data were selected as the control group, as detailed in S1 Table. To address potential biases in control group selection, the Propensity Score Matching—Difference in Differences (PSM-DID) method was employed to enhance the robustness of the results. This methodological approach allows for more accurate matching of cities in the experimental and control groups, thereby improving the validity of the causal inferences drawn from the study. The data for this research was primarily sourced from the ’China Statistical Yearbook’ and the ’China Urban Construction Statistical Yearbook.’ These comprehensive annual publications provide a wealth of reliable and detailed information relevant to urban development and municipal services in China. To address the issue of missing values within the dataset, a linear interpolation method was employed. This technique was chosen for its ability to estimate missing data points within a series, ensuring the continuity and completeness of the data set.

### 2.2. Research hypothesis

For the cities designated as pilot sites for MSW sorting, priority policy support is expected. It is hypothesized that, due to the targeted objectives set by these policies and the preferential support from relevant governmental departments, waste sorting implementation in pilot cities will be more effective compared to non-pilot cities. A key indicator of this effectiveness is the volume of urban household waste processed, which reflects a city’s capability for sustainable development. It is anticipated that pilot cities will show superior performance in this regard. Accordingly, this paper proposes the following specific research hypotheses, grounded in the data trends observed and the theoretical framework of urban sustainability.

**Hypothesis 1: The municipal solid waste (MSW) sorting pilot policy can significantly improve the sustainable development capacity**.

Furthermore, it is widely recognized that augmenting fixed asset investment in waste management infrastructure and improving citizens’ environmental awareness are key strategies for enhancing a city’s waste treatment capabilities and overall sustainability.

**Fig 1:**
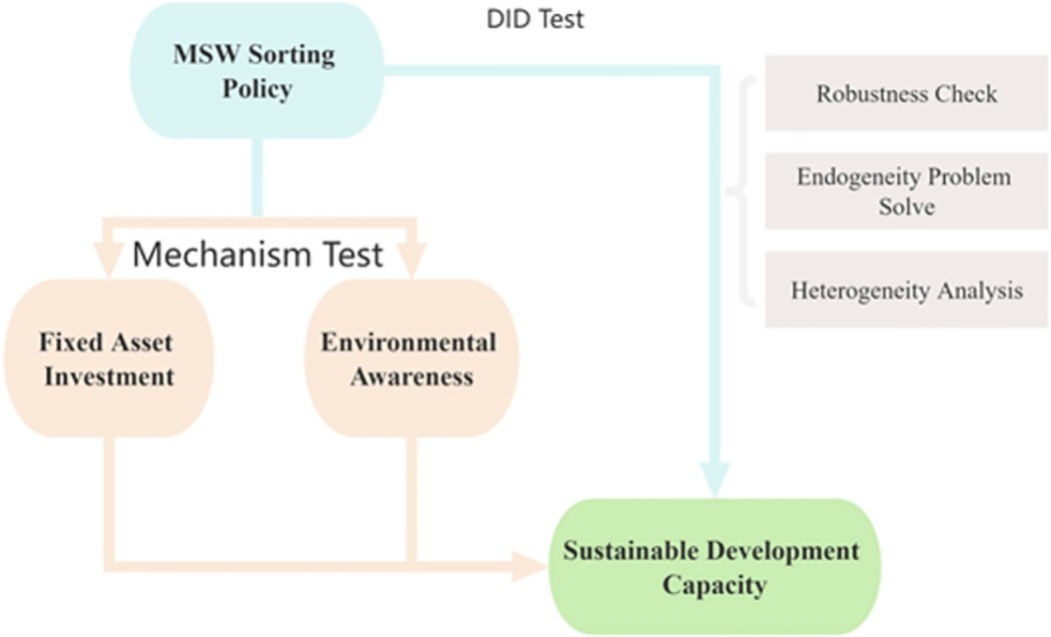
Main research methods and hypotheses.

Consequently, as Fig 1 shows, it is expected that pilot cities engaged in MSW sorting will enhance their waste management efficacy and sustainable development standards through these mechanisms. This leads to the formulation of the following additional hypotheses.

**Hypothesis 2: Pilot cities for MSW sorting can improve sustainable development capacity by increasing investment in fixed assets for waste treatment in their local areas**.
**Hypothesis 3: Pilot cities for MSW sorting can improve sustainable development capacity by increasing environmental awareness for waste treatment in their local areas**.

### 2.3. Research model

**Fig 2:**
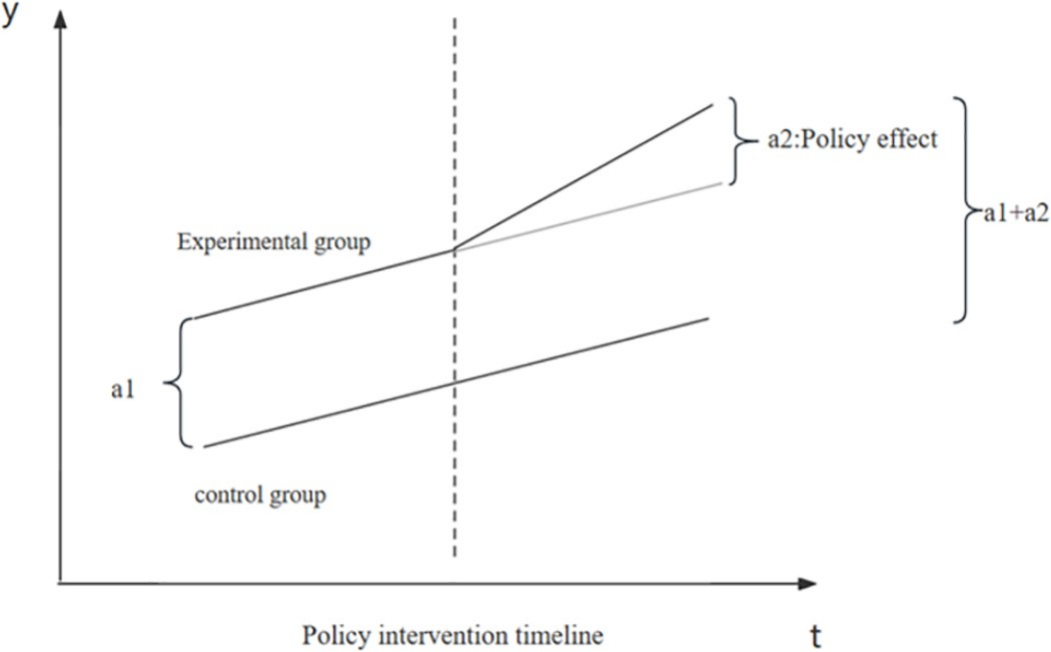
Diagram of Difference in Difference (DID) analysis.

This paper treats the pilot policy of municipal solid waste sorting as a quasi-natural experiment. It utilizes a difference-in-differences (DID) model to investigate the policy effects of municipal solid waste sorting pilots. As shown in Fig 2, the difference in the variable to be explained between the experimental group and the control group before the policy intervention is a1. After the intervention is applied to the experimental group, the difference in the variable to be explained between the experimental group and the control group becomes a1 + a2. Therefore, a2 is the effect of the policy intervention.

The specific DID model constructed is as follows:

$${\text{Capacity}}_{it}=\beta_{0}+\beta_{1}did_{it}+\beta_{2}X_{it}+\mu_{i}+\eta_{t}+\varepsilon_{it}$$ (1)

Among them, the subscripts i and t represent the city and year, respectively, and Capacity represents the dependent variable, namely the harmless treatment amount of MSW; Did represents the core explanatory variable, which is the dummy variable of the MSW sorting pilot policy; X represents a series of control variables; *μ*_*i*_ represents urban fixed effects, η_t_ represents year fixed effects, and *ε*_*it*_ represents random perturbation terms. This article clusters standard errors at the urban level to control the influence of autocorrelation, heteroscedasticity, and other factors in the model.

This study adopts the mediation effect model to identify further how the pilot policy of municipal solid waste sorting affects the sustainable development capacity. Specifically, based on model (1), the following two recursive models are constructed:

$$M_{it}=\varphi_{0}+\varphi_{1}did_{it}+\varphi_{2}X_{it}+\mu_{i}+\eta_{t}+\varepsilon_{it}$$ (2)

$$capacity_{it}=\gamma_{0}+\gamma_{1}did_{it}+\gamma_{2}M_{it}+\gamma_{3}X_{it}+\mu_{i}+\eta_{t}+\varepsilon_{it}$$ (3)

*M*_*it*_ is the mediating variable, representing the fixed asset investment and environmental awareness campaign intensity in municipal solid waste treatment of the city. The meanings of the other variables are the same as in model (1). The test of the mediation effect model is divided into three steps. Firstly, estimate the coefficient *β*_1_ of model (1) to examine the total effect of the municipal solid waste sorting pilot policy on the sustainable development capacity. If *β*_1_ is significantly positive; it indicates that the municipal solid waste sorting pilot policy can significantly improve the sustainable development capacity. Secondly, estimate the coefficients *φ*_1_ and *γ*_2_ of models (2) and (3), respectively. If both are significant, it suggests the presence of a mediation effect. Based on this, if the coefficient *γ*_1_ of model (3) is not significant, it means a full mediating role. If the coefficient *γ*_1_ of model (3) is significantly positive and less than the coefficient *β*_1_ of model (1), it indicates a partial mediating effect.

### 2.4. Variables

Dependent Variable: Harmless Treatment Volume of Municipal Solid Waste (capacity). The capability to handle Municipal Solid Waste (MSW) is an essential indicator of a city’s ability for sustainable development [15]. Thus, we use the harmless treatment volume of municipal solid waste as an indicator to measure the level of sustainability [16].

Key Explanatory Variable: MSW Sorting Pilot Policy Variable (did). In this paper, this policy is set up in the form of a dummy variable. Specifically, did is equal to the product of treat and time. Treat denotes an individual dummy variable: if it belongs to a city where the municipal solid waste sorting pilot policy is implemented, treat is set to 1; otherwise, it is set to 0. Time is a time dummy variable: if the year is 2017 or later when the municipal solid waste sorting pilot cities were determined, then time is set to 1; otherwise, it is set to 0.

Mediating Variable: (1) Municipal Solid Waste Treatment Fixed Asset Investment (investment). We use the municipal solid waste treatment fixed asset investment as a measure of the emphasis on the waste sorting policy in pilot cities. A higher investment indicates that waste sorting is given more attention in the city. (2) Environmental Awareness (awareness). Utilizing the keyword "MSW," retrieve pertinent policy documents and employ web scraping techniques to collect and quantify the number of policies that include the keyword "promotional education." A higher count of policy instances indicates a stronger emphasis on environmental awareness campaigns.

Control Variables: Based on the existing research, in the empirical analysis, this study primarily controls the following variables: the proportion of the tertiary industry in GDP (X1) [17], the level of economic development (X2) [18], number of MSW facilities (Transportation Facilities (X3) [19], treatment facilities (X4) [20], education (X5) [21], environmental expenditure (X6) [22], population density (popdense (X7) [21]. The definitions and statistical descriptions of variables are presented in Tables 1 and 2, respectively.

**Table 1:** Explanations for variables in regressions.

Variables		Code	Data Source	Unit
Dependent Variable:	MSW Disposal Volume	Capacity	China Urban Construction Statistical Yearbook	10,000 tons
Explanatory Variable	MSW sorting Policy	did	The pilot policy list	
Control Variables	Proportion of Tertiary Industry in GDP	X_1_	China City Statistical Yearbook	%
	The level of economic development	X_2_		Yuan
	MSW Transportation Facilities	X_3_	China Urban Construction Statistical Yearbook	Units
	MSW Treatment Facilities	X_4_		Sites
	Education	X_5_	China City Statistical Yearbook	People
	Environmental Expenditure	X_6_		Yuan
	Population Density	X_7_		People/square kilometer
Mediating Variable	Fixed Asset Investment in MSW	Investment	China Urban Construction Statistical Yearbook	10,000 Yuan
	Environmental Awareness	Awareness	Collecting from the Internet	

**Table 2:** Descriptive statistics of variables.

Variable Name	Observations	Mean	Standard Deviation	Minimum	Maximum
capacity	1425	1.551	0.431	0.041	3.088
did	1425	0.053	0.225	0.000	1.000
X_1_	1425	7.920	1.701	1.609	12.720
X_2_	1425	0.253	0.297	0.000	1.531
X_3_	1425	4.597	0.357	3.441	5.577
X_4_	1425	0.412	0.097	0.000	0.729
X_5_	1425	2.370	0.574	0.845	3.903
X_6_	1425	4.917	1.096	0.637	7.708
X_7_	1425	8.686	0.792	6.305	10.743

## 3. Empirical results

### 3.1. Benchmark regression results

The impact of the MSW sorting pilot policy on sustainable development capacity is detailed in Table 3. In the initial model (column 1), without the inclusion of any control variables, the estimated coefficient for the ’difference-in-differences’ (DID) variable is 0.576, significant at the 1% level. This preliminary result suggests that the pilot policy has positively influenced sustainable development capacity. The regression results from columns (2) to (8), which include a series of different control variables, consistently show that the DID coefficients remain significantly positive and stable, passing the 10% significance threshold. This consistency reinforces the conclusion that the MSW sorting pilot policy has indeed enhanced sustainable development capacity, thereby supporting Hypothesis 1Regarding the control variables in our model, the estimate for variable X1, representing the development of the tertiary sector, shows a significantly positive effect, suggesting an optimistic influence on the advancement of household waste treatment technology and the city’s capacity for sustainable development. The statistical significance of this variable aligns with the findings of Ding et al. [23], who observed similar positive correlations in their research. This parallel not only validates our results but also highlights the critical role of tertiary sector development in enhancing urban sustainability and waste management efficiency.

**Table 3:** Benchmark regression results.

Variables	(1)	(2)	(3)	(4)	(5)	(6)	(7)	(8)
did	0.576*** (0.054)	0.233*** (0.049)	0.155*** (0.043)	0.128*** (0.043)	0.079* (0.042)	0.086** (0.041)	0.090** (0.041)	0.090** (0.041)
X_1_		0.031*** (0.001)	0.014*** (0.001)	0.012*** (0.002)	0.010*** (0.002)	0.010*** (0.002)	0.009*** (0.002)	0.009*** (0.002)
X_2_			0.455*** (0.021)	0.360*** (0.028)	0.320*** (0.027)	0.299*** (0.028)	0.154** (0.063)	0.154** (0.063)
X_3_				0.098*** (0.019)	0.085*** (0.019)	0.091*** (0.018)	0.087*** (0.018)	0.087*** (0.018)
X_4_					0.212*** (0.025)	0.206*** (0.024)	0.208*** (0.024)	0.208*** (0.024)
X_5_						0.017 (0.016)	0.012 (0.016)	0.012 (0.016)
X_6_							0.117** (0.046)	0.117** (0.046)
X_7_								0.006 (0.024)
Constant	3.526*** (0.011)	2.268*** (0.061)	-1.856*** (0.199)	-1.283*** (0.227)	-0.817*** (0.228)	-0.693*** (0.224)	-0.103 (0.322)	-0.148 (0.367)
N	1425	1425	1425	1425	1425	1425	1425	1425
R^2^	0.080	0.308	0.486	0.496	0.538	0.540	0.543	0.543

Note:

*, **, and *** respectively indicate significance at the 10%, 5%, and 1% levels; numbers inside parentheses represent standard errors.

The estimated coefficient for control variable X2 is significantly positive at the 5% level, suggesting that improvements in economic development substantially enhance MSW processing capabilities and urban sustainability. This finding corroborates Liu’s research [18], which similarly highlights the positive impact of economic growth on sustainable waste management.

The coefficient for X3 is significantly positive, indicating that an increase in the number of vehicles enhances MSW collection and transportation efficiency, thereby contributing to the city’s sustainable development. This result is in alignment with the studies conducted by Fan and Gu [19,24], who also noted the positive influence of transportation infrastructure on waste management.

The estimated coefficient for X4 is significantly positive at the 1% significance level, indicating that the establishment of MSW treatment facilities has effectively improved the level of MSW processing and further enhanced the city’s capacity for sustainable development. This conclusion is consistent with the findings of Bezama [20].

The estimated coefficient for X6 is significantly positive at the 5% significance level, indicating that an increase in fiscal expenditure has improved the city’s capacity for sustainable development. This supports the broader sustainable development theory [22], underlining the role of financial resources in advancing sustainability goals.

### 3.2. Robustness check

To ensure the reliability of the benchmark regression results, this study will conduct a robustness check on the baseline regression model from three aspects.

#### 3.2.1. PSM-DID estimation

During the implementation of the MSW sorting pilot policy, the selection of pilot cities may exhibit selection bias, as cities with more advanced economic development and comprehensive infrastructure are more likely to be chosen. This could lead to biases in the baseline regression results. Therefore, this study further estimates the model using the PSM-DID method. Specifically, this study uses the volume of harmless treatment of household waste as the outcome variable and the control variables in the baseline model as covariates. We employ 1:1 neighbor matching, radius matching, and kernel matching methods for matching. The results show that the bias between the compared pilot and non-pilot cities in waste sorting significantly decreases. Moreover, there is no significant difference in all covariates at the 10% level, indicating good matching performance.

Based on the matched sample, the study re-estimates the impact of the MSW sorting pilot policy on sustainable development capacity, as detailed in columns (1), (2), and (3) of Table 4. The results reveal that the estimated coefficient for the ’did’ variable is significantly positive at the 10% level, reinforcing the positive impact of the MSW sorting pilot policy on sustainable development capacity.

**Table 4:** PSM-DID results.

Variables	1:1 Neighbor Matching	Radius Matching	Kernel Matching
	(1)	(2)	(3)
did	0.067* (0.034)	0.062*** (0.019)	0.054*** (0.019)
Control Variables	Yes	Yes	Yes
Constant	1.179*** (0.402)	-0.684*** (0.126)	-0.634*** (0.142)
N	90	1095	945
R^2^	0.609	0.489	0.464

Note:

*, **, and *** respectively indicate significance at the 10%, 5%, and 1%levels; numbers inside parentheses represent standard errors.

#### 3.2.2. Placebo test

**Fig 3:**
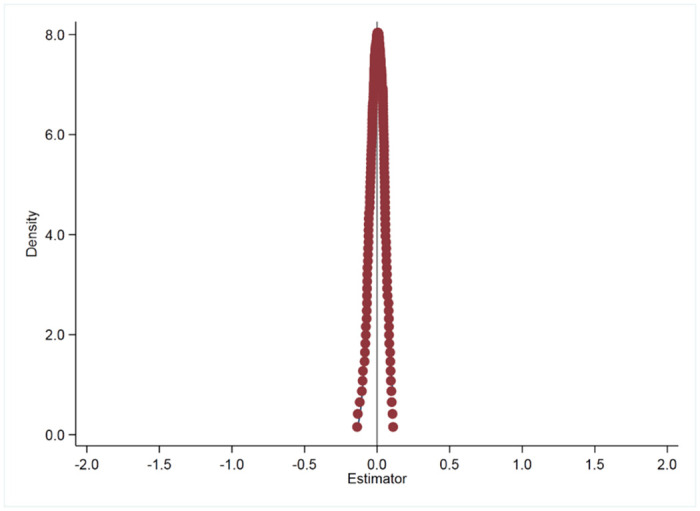
Placebo looped 200 times.

**Fig 4:**
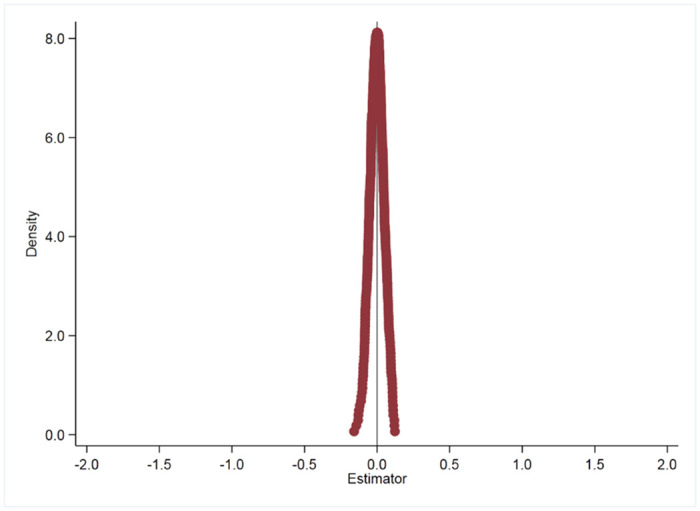
Placebo looped 500 times.

To mitigate the potential influence of random factors on regression results, this study adopts a placebo testing method inspired by Cantoni et al. (2017) [25]. In this approach, cities were randomly selected in numbers equal to the actual pilot cities for solid waste sorting, forming a hypothetical treatment group. A surrogate policy metric was then applied to the baseline regression model. This process was repeated 200 and 500 times to generate a robust sample. The resulting kernel density visualizations of the approximated coefficients from these 200 and 500 regressions are presented in Figs 3 and 4. The visualizations reveal that the coefficient distribution across these iterations closely approximates a normal distribution, indicating the non-randomness of the effect. This pattern suggests that the observed impact of the MSW sorting pilot policy on sustainable development capability is not likely to be attributed to external random variables, thereby strengthening the validity of the foundational regression estimations in this study.

To ensure the rigorousness of the study, robustness checks were conducted through four distinct approaches:

1. Temporal Sample Refinement: The study originally covered the period from 2006 to 2020. Considering the inception of the pilot policy in 2017, the analysis was re-evaluated using a narrowed timeframe of 2014–2020. This focused approach examines the effects three years before and after the policy implementation, reducing potential biases related to the chronological span of the sample.
2. Control for Time-varying Policy Effects: To address the impact of varying policy effects over time and random factors in different cities, the study incorporated combined fixed effects for cities and years, followed by re-estimation of the model.
3. Outlier Rectification: The study also adjusted for outliers by truncating the dependent variable at the 1% level, ensuring the results are not skewed by extreme values.
4. Differentiated city stratification: Recognizing the differences in waste classification between municipalities directly under the central government (Beijing, Shanghai, Tianjin, Chongqing) and other administrative levels, additional estimates were made after excluding cities that may have significant differences from the overall sample. This exclusion is pivotal for ensuring that the findings are not overly influenced by the unique waste management frameworks and infrastructural capabilities of these major urban centers. By focusing on a broader range of cities, the analysis seeks to provide insights that are more representative of diverse urban contexts across the country, thereby enhancing the generalizability of the study’s conclusions.

The results of the above series of robustness tests are shown in columns (1) to (4) of Table 5. The results indicate that the estimated coefficients of all models are significantly positive, further supporting the baseline regression results of this study.

**Table 5:** Robustness test results.

Variables	2014–2020	Joint Fixed Effects	Remove Outliers	Exclude Municipalities
	(1)	(2)	(3)	(4)
did	0.055*** (0.016)	0.046** (0.022)	0.068*** (0.021)	0.070*** (0.023)
Control Variables	Yes	Yes	Yes	Yes
Constant	0.398* (0.214)	0.984*** (0.255)	-0.441*** (0.111)	-0.533*** (0.118)
Observations	665	1425	1425	1395
R2	0.391	0.434	0.406	0.395

Note:

*, **, and *** respectively indicate significance at the 10%, 5%, and 1 levels; numbers inside parentheses represent standard errors.

### 3.3. Endogeneity problem

The benchmark regression results, in tandem with the conducted robustness checks, offer preliminary support for the hypothesis that the MSW sorting pilot policy enhances urban sustainable development. However, a potential concern of endogeneity in these conclusions cannot be overlooked. To address this complexity, the study progresses by employing the Two-Stage Least Squares (2SLS) methodology. This approach is utilized as an instrumental variable regression paradigm, specifically designed to mitigate potential endogeneity issues. By applying 2SLS, the study aims to provide more robust and reliable conclusions, further solidifying the initial validation of the research proposition. We chose the "number of cities with solid waste sorting pilots within a province divided by the total number of other cities in the province (peer)" as the instrumental variable for the pilot policy on MSW sorting. Table 6 presents the results of the instrumental variable regression using the Two-Stage Least Squares (2SLS) method. The F-statistic from the first-stage deterioration exceeds the empirical threshold of 10, indicating that the constructed instrumental variable is highly correlated with the endogenous explanatory variable. Additionally, the null hypothesis of insufficient instrument identification in the Anderson canon. Corr. LM test is rejected, suggesting that the instrumental variable is related to the endogenous explanatory variable. The results also indicate no weak instrument problem. The second-stage regression results show that, whether or not control variables are included, the core explanatory variable’s coefficient is significant at the 1% level, further indicating that the pilot policy on MSW sorting significantly improves the sustainable development capacity in cities. It can be seen that even considering issues of endogeneity arising from potential reverse causality, the core conclusions of this study remain robust and reliable.

**Table 6:** Endogeneity test.

Variable	Using Peer as an Instrumental Variable	
	(1)	(2)
did	1.099*** (0.112)	0.297*** (0.100)
Control Variables	No	Yes
Constant	1.493*** (0.012)	-0.146 (0.099)
N	1425	1425
R2	0.097	0.684
1st F	326.000	160.770
Anderson-Rubin Wald	93.020***	9.810***
Stock-Wright LM S	87.320***	9.740***
Anderson canon. corr. LM	265.612***	145.020***
Weak identification test	326.005 (16.38)	160.771 (16.38)

Note:

*, **, and *** indicate significance at the 10%, 5%, and 1% levels, respectively. Standard errors are reported in parentheses.

### 3.4. Mechanism test

The previous section indicates that the pilot policy for MSW sorting can significantly enhance the sustainable development level of cities. However, the underlying mechanism of its effectiveness remains unclear.

Table 7 reports the potential mechanisms by which pilot policies on urban household waste enhance urban sustainable development. The results show that ’investment’ passes the 1% significance test, with a coefficient of 0.586 and a standard error of 0.105. ’Awareness’ did not pass the 10% significance test. By increasing fixed asset investment in local household waste treatment, pilot cities for waste sorting can improve their capacity for sustainable development. This validates Hypothesis 2 and rejects Hypothesis 3. These findings also support the research conclusions of [11].

**Table 7:** Mechanism test results.

Potential Mediating Variables	Awareness	Investment
Mediating Test	Not Pass	Pass
Control Variables	Yes	Yes
Test Model	Model (2) and Model (3)	

Note: *, **, and *** indicate significance at the 10%, 5%, and 1% levels, respectively. Standard errors are reported in parentheses.

### 3.5. Heterogeneity analysis

Given China’s vast territory and significant differences in geographical location, economic development level, and population distribution, the municipal solid waste sorting pilot policy’s impact on sustainable development capacity may differ across cities. This section analyzes the heterogeneous effects of the pilot policy based on the city’s geographic location, economic scale, and population density.

Firstly, the heterogeneity analysis focused on urban geographical location, categorizing cities into eastern, central, and western regions according to the classification standards set by the National Development and Reform Commission. The specific regression results are delineated in Table 8. In terms of geographical location, the DID estimation coefficients for cities in both eastern and central regions were found to be insignificant. However, in the western region, the DID coefficients are significantly positive at the 1% level, and notably higher in magnitude. This indicates that the solid waste sorting pilot policy has a more pronounced policy effect in the western region, significantly enhancing its capacity for sustainable development. This regional disparity highlights the importance of tailoring policy interventions to the specific socio-economic and geographical contexts of different regions.

**Table 8:** Heterogeneity test: Location.

Variable	East	Central	West
	(1)	(2)	(3)
did	-0.039 (0.025)	0.055 (0.036)	0.270*** (0.047)
Control Variable	Yes	Yes	Yes
Constant	-0.372*** (0.143)	-0.222 (0.193)	-0.985*** (0.243)
N	645	435	345
R^2^	0.639	0.425	0.420

Note:

*, **, and *** indicate significance at the 10%, 5%, and 1% levels, respectively. Standard errors are reported in parentheses.

Secondly, the study conducted a heterogeneity analysis based on the urban economic scale. By calculating the average per capita GDP of each city over the study period and ranking the data, cities were divided into two groups: high and low economic scales, based on the median value. This division was employed as the criterion for categorizing urban economic scales. The heterogeneous analysis on these segmented samples yielded specific regression results, as illustrated in Table 9. Irrespective of the economic scale, the estimated ’difference-in-differences’ (DID) coefficient is significantly positive at the 5% level. Notably, the coefficient for regions with a lower economic scale is 0.121, which is significantly higher than the coefficient of 0.05 for areas with a higher economic scale. This suggests that the policy impacts of the solid waste sorting pilot program are more substantial in regions with relatively lower levels of economic development, highlighting the importance of targeting policy measures in cities based on their economic profiles.

**Table 9:** Heterogeneity test: Economic scale.

Variable	High	Low
	(1)	(2)
did	0.050** (0.023)	0.121** (0.049)
Control Variable	Yes	Yes
Constant	-0.952*** (0.170)	-0.218 (0.151)
N	705	720
R^2^	0.521	0.373

Note:

*, **, and *** indicate significance at the 10%, 5%, and 1% levels, respectively. Standard errors are reported in parentheses.

Thirdly, the study examined the impact of urban population density on the effectiveness of the solid waste sorting pilot policy. Utilizing the median value of average population density for each city over the study period, the sample was divided into two categories: high-density and low-density urban areas. The heterogeneous analysis on these segmented samples yielded specific regression results, as detailed in Table 10. In regions with high population density, the estimated ’difference-in-differences’ (DID) coefficient is significantly positive at the 1% level, indicating a strong policy effect. In contrast, the coefficient for areas with lower population density is found to be insignificant. This disparity suggests that the pilot program for solid waste sorting exhibits more pronounced effects in areas with higher population density. This finding underscores the importance of considering urban population density in the formulation and implementation of waste management policies.

**Table 10:** Heterogeneity test: Population density.

Variable	High	Low
	(1)	(2)
did	0.100*** (0.028)	0.047 (0.031)
Control Variables	Yes	Yes
Constant	-0.669*** (0.170)	-0.156 (0.146)
N	705	720
R^2^	0.434	0.470

Note:

*, **, and *** indicate significance at the 10%, 5%, and 1% levels, respectively. Standard errors are reported in parentheses.

The findings of this research underscore the necessity of tailoring policy implementation to align with the varied economic development levels and population sizes across different regions. These insights highlight the importance of customizing waste management strategies to optimize policy effectiveness, taking into account regional economic and demographic characteristics. Such an approach ensures that policies are not only more contextually relevant but also more likely to achieve the desired outcomes in enhancing sustainable waste management practices.

## 4. Discussion

The verification of Hypothesis 1 elucidates the relationship between the pilot policy on urban solid waste sorting and sustainable development capabilities. Our findings are consistent with the research results of Guerrero et al. [15], which further underscores the necessity for implementing urban solid waste sorting policies. In fact, according to the research of Sebos et al. [26,27], the processing capacity of Municipal Solid Waste (MSW) is related to greenhouse gas emissions, which in turn can impact the capacity for sustainable development.

Based on the estimated results of the control variables, first, the development of the tertiary sector has promoted the advancement of household waste treatment technology and the city’s capacity for sustainable development. This is consistent with the findings of Ding et al. [23]. This also supports that a shift in human activity patterns can protect the natural environment and contribute to sustainable development [28].

Second, improving economic development dramatically enhances MSW processing capabilities and the city’s capacity for sustainable development. This conclusion is supported by Liu’s research as well [18]. However, Sebos et al thought that an increase in the level of economic development does not necessarily imply an enhancement in sustainability capacity [29], as the implementation of public policies requires a consensus among stakeholders.

Third, increasing the number of vehicles has improved the level of MSW collection and transportation, laying the foundation for enhancing the city’s capacity for sustainable development. This aligns with Fan’s and Gu’s research [19,24]. It is noteworthy that, according to Papadogiannaki’s study during COVID-19 [30], an increase in vehicle numbers also leads to higher levels of air pollution. Therefore, it is essential to green and decarbonize transportation, build green infrastructure, such as using electric trucks, and design more efficient collection routes in urban planning. Additionally, adopting digital means, such as remotely operating robots and drones, can reduce the carbon footprint. Thus, a multi-sectoral interactive collaboration is indispensable [31]. By fostering group cooperation among different stakeholders, sustainable development can be more effectively achieved.

Fourth, the establishment of MSW treatment facilities has effectively improved the level of MSW processing and further enhanced the city’s capacity for sustainable development. This conclusion is consistent with the findings of Bezama [20].

Fifth, increased fiscal expenditure has improved the city’s ability for sustainable development. This finding supports sustainable development theory [22]. This also confirms that the implementation of public policies cannot be separated from the support of financial resource [29].

The results of the mechanism analysis support Hypothesis 2 and reject Hypothesis 3, indicating that the improvement in MSW (Municipal Solid Waste) treatment capabilities will be more pronounced if accompanied by an increase in fixed asset investment. For cities with limited fiscal budgets, more attention should be given to fixed asset investments. Considering the better implementation of policies, pilot policies can be further subdivided to differentiate the intensity of policy measures, thus enhancing the effectiveness of the policies [32]. Many pilot cities have shown impressive performance in fixed asset investment, which has also boosted local green and low-carbon transformation. Taking Beijing as an example, the city began replacing its diesel-powered trucks with new energy smart trucks in 2020. These newly equipped garbage collection vehicles are fitted with weighing devices and cameras, allowing for the entire process of waste collection and transportation to be recorded and uploaded to a command center. Additionally, in 2022, all 4,274 garbage collection vehicles in the city were repainted: food waste vehicles in green, other waste in gray, and recyclable materials in blue. By 2022, Beijing had built 63,500 fixed bin stations, 2,095 sorting stations, and 138 transfer stations. With these facilities, the city achieved a 78% resource utilization rate for household waste and a 38% recycling rate.

Although public environmental awareness is not a significant mediator variable, according to the research of Zerva et al. [13], it is necessary to strengthen public environmental awareness. Across various regions, particularly those with lower population density and economic development, enhancing public awareness of waste classification and sustainability is vital. This could be achieved through educational campaigns and community initiatives.

Urban solid waste disposal possesses potential carbon emission reduction attributes [33], and the MSW (Municipal Solid Waste) sorting pilot policy is also a part of China’s ambitious carbon neutrality plan. By increasing the recycling rate of recyclables and the capacity of MSW disposal, it is possible to maximize the carbon reduction benefits throughout the entire process of urban solid waste disposal, aiding in the achievement of carbon neutrality goals [34]. Therefore, it is necessary to incorporate MSW sorting policies into the public policy objectives of cities, establish a broader social consensus, and promote better policy implementation [13].

## 5. Conclusions and policy implications

This study assesses the impact of the Municipal Solid Waste (MSW) sorting pilot policy on urban sustainable development. Utilizing a Difference-in-Differences (DID) model, the paper provides insights into the policy’s effectiveness. Key conclusions are:

1. Empirical evidence demonstrates that the MSW sorting pilot policy significantly enhances urban sustainable development capacity. This finding is particularly relevant for developing countries, offering a practical approach to waste management challenges.
2. Analysis of mediation effects reveals that in developing regions, particularly those with pressing MSW issues, investment in fixed assets yields more substantial benefits compared to awareness and education campaigns.
3. Heterogeneity analysis advises against a universal policy application. Instead, it suggests tailoring policies to align with specific city characteristics, such as economic status and population size. For example, Western regions may require more investment and technical support for waste classification and treatment, while densely populated areas could adopt more proactive waste management policies.

According to the World Bank [35], in low-income countries, over 90% of waste is often dumped in unregulated landfills or openly burned. In 2020, approximately 2.24 billion tons of solid waste were generated globally, projected to increase by 73% by 2050. This study suggests through mediation effect analysis that international organizations and green investment institutions should prioritize investments in local waste classification and processing equipment. Compared with previous studies, this article provides a more feasible approach to address the waste management challenges in financially constrained environments.

## Supporting information

S1 Table Pilot cities and control groups. (XLSX)

S1 Data Supporting data for DID. (XLSX)
